# nGASP – the nematode genome annotation assessment project

**DOI:** 10.1186/1471-2105-9-549

**Published:** 2008-12-19

**Authors:** Avril Coghlan, Tristan J Fiedler, Sheldon J McKay, Paul Flicek, Todd W Harris, Darin Blasiar, the nGASP Consortium, Lincoln D Stein

**Affiliations:** 1 Wellcome Trust Sanger Institute, Wellcome Trust Genome Campus, Hinxton, Cambridge, CB10 1SA, UK; 2 Department of Biological Sciences, Florida Institute of Technology, Melbourne, FL 32901, USA; 3 Cold Spring Harbor Laboratory, Cold Spring Harbor, NY 11724, USA; 4 European Bioinformatics Institute, Wellcome Trust Genome Campus, Hinxton, Cambridge, CB10 1SD, UK; 5 Washington University School of Medicine, St Louis, MO 63108, USA

## Abstract

**Background:**

While the *C. elegans *genome is extensively annotated, relatively little information is available for other *Caenorhabditis *species. The nematode genome annotation assessment project (nGASP) was launched to objectively assess the accuracy of protein-coding gene prediction software in *C. elegans*, and to apply this knowledge to the annotation of the genomes of four additional *Caenorhabditis *species and other nematodes. Seventeen groups worldwide participated in nGASP, and submitted 47 prediction sets across 10 Mb of the *C. elegans *genome. Predictions were compared to reference gene sets consisting of confirmed or manually curated gene models from WormBase.

**Results:**

The most accurate gene-finders were 'combiner' algorithms, which made use of transcript- and protein-alignments and multi-genome alignments, as well as gene predictions from other gene-finders. Gene-finders that used alignments of ESTs, mRNAs and proteins came in second. There was a tie for third place between gene-finders that used multi-genome alignments and *ab initio *gene-finders. The median gene level sensitivity of combiners was 78% and their specificity was 42%, which is nearly the same accuracy reported for combiners in the human genome. *C. elegans *genes with exons of unusual hexamer content, as well as those with unusually many exons, short exons, long introns, a weak translation start signal, weak splice sites, or poorly conserved orthologs posed the greatest difficulty for gene-finders.

**Conclusion:**

This experiment establishes a baseline of gene prediction accuracy in *Caenorhabditis *genomes, and has guided the choice of gene-finders for the annotation of newly sequenced genomes of *Caenorhabditis *and other nematode species. We have created new gene sets for *C. briggsae*, *C. remanei*, *C. brenneri*, *C. japonica*, and *Brugia malayi *using some of the best-performing gene-finders.

## Background

The promise of comparative genomics among the nematodes has motivated sequencing in *Caenorhabditis elegans*, *C. briggsae*, *C. brenneri*, *C. remanei*, and *C. japonica *[1-3]. While the *C. elegans *genome has been extensively annotated, relatively little information is available for the other *Caenorhabditis *genomes [4]. In addition, the genome of the distantly related nematode *Brugia malayi *was recently published [5], and those of many other nematodes are currently being sequenced such as *Pristionchus*, *Haemonchus*, *Meloidogyne*, and *Trichinella*. An essential step in the analysis of these genomes will be to identify and annotate their protein-coding genes, but it is not known which gene prediction systems perform best on nematode genomes. To address this issue, the nematode genome annotation assessment project (nGASP) was launched to assess the accuracy of protein-coding gene prediction software in *C. elegan*s, and then to apply this knowledge to annotating other *Caenorhabditis *genomes.

The nGASP project parallels recent computational prediction initiatives including CASP for protein structure prediction [6], GASP for *Drosophila *gene prediction [7], and EGASP for human gene prediction [8]. Scientists working in the field of computational gene prediction were invited to participate in nGASP. Participants were provided with training and test sets, each comprising ten non-overlapping 1-Mb genomic sequence regions, representing ~10% of the *C. elegans *genome. We also provided auxiliary data to the participants to use for training their gene-finders. The auxiliary data included multi-genome alignments between *C. elegans*, *C. briggsae *and *C. remanei*, and alignments of ESTs, mRNAs and proteins to the *C. elegans *genome.

nGASP was conducted in two phases. The first phase of the competition was open to all gene prediction programs and was divided into three categories: category 1 predictions were based on genomic sequence alone (*ab initio *gene-finders); category 2 was open to gene-finders that use nucleotide level multi-genome alignments; and category 3 predictions encompassed gene-finders that take advantage of alignments of expressed sequences such as proteins, ESTs, and assembled mRNAs. After the first phase of the competition was complete, we posted the output of each of the predictors to the nGASP web site . We then began phase two of the competition, which was open to 'combiners' (category 4), defined as gene prediction systems that use gene models created by other annotation software, and any of the data used as input for the phase one gene-finders. To assess the accuracy of the submitted predicted gene sets, we quantified their sensitivity and specificity in predicting coding regions by using the metrics from GASP [7] and EGASP [8]. Here, we describe the performance of the most accurate gene-finders in *C. elegans*, identify some common features of *C. elegans *genes that the majority of gene-finders find hard to predict correctly, and discuss the choice of gene predictors for the annotation of the newly sequenced genomes of other nematode species.

## Results and discussion

### Submitted gene sets

Seventeen groups worldwide participated in nGASP, and submitted 47 prediction sets for 10 Mb of the *C. elegans *genome (Table 1). Several groups submitted predicted gene sets for more than one category, or more than one entry per category generated by running their programs under different parameter sets. The submitted gene sets, and the details of the parameters used to make them, are available on the nGASP ftp site .

**Table 1:** Participating groups and submitted gene sets.

**Participating group**	**Program name**	**Number of gene sets submitted in each category**
Blasiar et al, Saint Louis, USA	GESECA (D. Blasiar, unpublished)	cat4:1

Borodovsky et al, Atlanta, USA	GeneMark.hmm [24]	cat1:1

Brent et al, Saint Louis, USA	N-SCAN [25]	cat2:1

Durbin et al, Cambridge, UK	GENOMIX [26]	cat4:2

Guigó et al, Barcelona, Spain	GeneID^1 ^[27], SGP2 [28]	GeneID: cat1:1, cat4:2; SGP2: cat2:1

Korf et al, Davis, USA	SNAP [29]	cat1:1

Krogh et al, Copenhagen, Denmark	Agene [30]	cat1:1

Liang et al, Cold Spring Harbor, USA	Gramene (Liang et al, unpublished)	cat3:2, cat4:1

Pereira et al, Pennsylvania, USA	Evigan [31], CRAIG [32]	CRAIG: cat1:1; Evigan: cat4:1

Rätsch et al, Tübingen, Germany	MGENE (Schweikert et al, submitted)	cat1:3, cat2:2, cat3:3

Roos et al, Pennsylvania, USA	GLEAN [33]	cat4:1

Salzberg et al, Maryland, USA	JIGSAW [14], GlimmerHMM [14]	GlimmerHMM: cat1:1; JIGSAW: cat4:2

Schiex et al, Toulouse, France	EUGENE [34]	cat1:1, cat2:1, cat3:2, cat4:4

Solovyev et al, University of London and Softberry Inc, New York, USA	Fgenesh, Fgenesh++, Fgenesh++C [13]	Fgenesh: cat1:1; Fgenesh++: cat3:1; Fgenesh++C: cat4:1

Stanke, Santa Cruz, USA	AUGUSTUS [12]	cat1:2, cat3:1

Brejová & Vinar, New York, USA	ExonHunter [35]	cat1:1, cat3:2

Yandell et al, Berkeley, USA	MAKER (using SNAP) [36]	cat3:2

^1^The GeneID gene set was submitted after the nGASP deadline. The research groups that participated in nGASP, the names of the software used to produce gene prediction sets, and the number of gene sets submitted in each of the nGASP categories by a research group, are given. There were four nGASP categories: category 1 predictions were made by *ab initio *gene-finders; category 2 predictions by gene-finders that use multi-genome alignments; category 3 predictions by gene-finders that take advantage of EST/mRNA or protein alignments; and category 4 predictions by gene prediction systems that use gene models created by other annotation software, and any of the data used as input for gene-finders in the other three categories. Here 'cat3:2' means that 2 gene sets in category 3 were submitted. In some cases a group submitted two gene sets produced by using different parameters of their software to the same nGASP category.

### Procedure for evaluation of gene-finding accuracy

The 10-Mb of test DNA sequence consisted of ten non-overlapping 1-Mb genomic regions of the *C. elegans *genome (Table 2). The gene predictions submitted to nGASP were evaluated using two reference gene sets drawn from WormBase (release WS160): (i) **ref1**, a 'sensitivity/accuracy' set consisting of genes from the test regions that were supported by full-length cDNAs across their entire coding region, and (ii) **ref2**, a 'full set' that contained all manually curated genes from the test regions (see **Methods**). The **ref2 **set includes both confirmed and unconfirmed gene models. Most of the unconfirmed gene models were initially based on predictions from the Genefinder software (P. Green, unpublished), but most of these have since been changed by manual curators on the basis of experimental data. nGASP differed from the *Drosophila *GASP [7] and human EGASP [8], in that curated gene structures for *C. elegans *were already publicly available, but participants were requested to not consult WormBase, GenBank or other databases for the curated gene models in the test regions.

**Table 2:** The nGASP test and training genomic regions.

**Type of nGASP region**	**Criterion used for selecting region**	**Coordinates in the *C. elegans *WS160 genome**
Training	High conservation, high gene density, autosomal	II: 2000001–3000000
Training	High conservation, high gene density, autosomal	V: 9000001–10000000
Training	High conservation, low gene density, autosomal	III: 1000001–2000000
Training	High conservation, low gene density, autosomal	IV: 2000001–3000000
Training	Low conservation, high gene density, autosomal	I: 12000001–13000000
Training	Low conservation, high gene density, autosomal	V: 4000001–5000000
Training	Low conservation, low gene density, autosomal	I: 2000001–3000000
Training	Low conservation, low gene density, autosomal	II: 13000001–14000000
Training	High conservation, low gene density, X-chromosome	X: 3000001–4000000
Training	High conservation, low gene density, X-chromosome	X: 2000001–3000000
Test	High conservation, high gene density, autosomal	IV: 7000001–8000000
Test	High conservation, high gene density, autosomal	V: 12000001–13000000
Test	High conservation, low gene density, autosomal	IV: 1–1000000
Test	High conservation, low gene density, autosomal	I: 14000001–15000000
Test	Low conservation, high gene density, autosomal	V: 16000001–17000000
Test	Low conservation, high gene density, autosomal	II: 1–1000000
Test	Low conservation, low gene density, autosomal	IV: 14000001–15000000
Test	Low conservation, low gene density, autosomal	I: 1000001–2000000
Test	High conservation, low gene density, X-chromosome	X: 4000001–5000000
Test	High conservation, low gene density, X-chromosome	X: 8000001–9000000

The ten 1-Mb regions of the *C. elegans *genome provided to the nGASP participants for training their gene-finders, and ten 1-Mb test regions in which they were asked to make gene predictions for the nGASP assessment.

We assessed sensitivity (Sn) using the **ref1 **reference and specificity (Sp) using the **ref2 **reference. Here sensitivity is defined as the proportion of real features (coding nucleotides, exons or genes) that have been correctly predicted in a particular gene set, while specificity is the proportion of predicted features in that gene set that correspond to real features. Note that the metric Sp is referred to as the positive predictive value or precision by statisticians, but consistent with previous work in the gene prediction field [7-9] we use the term 'specificity'. For each submitted gene set, we assessed its ability to accurately predict protein coding regions at the base, exon, isoform and gene levels, following the sensitivity and specificity definitions above, which were also used by EGASP [8]. The least stringent metrics were base level sensitivity and specificity, which measure whether a gene predictor is able to correctly classify a base as coding. Exon level metrics measure the ability of a gene prediction system to identify the exact left and right borders of the protein-coding regions of exons in the reference sets. By 'exon', we mean the protein-coding part of an exon (also known as the CDS, or coding sequence). Isoform level accuracy is the most stringent test. One *C. elegans *gene can produce several alternative spliced transcripts. For the purposes of nGASP we considered only the protein-coding portion of a transcriptional isoform, and scored a correctly predicted isoform if the protein-coding portions of all its exons were predicted accurately and no extra full or partially protein-coding exons were predicted. The gene level assessment of accuracy was intermediate in stringency between the exon and isoform levels. To be scored correct at the gene level, a gene predictor had to call at least one of the gene's isoforms correctly.

### Results from evaluation of gene-finding accuracy

**Figure 1:**
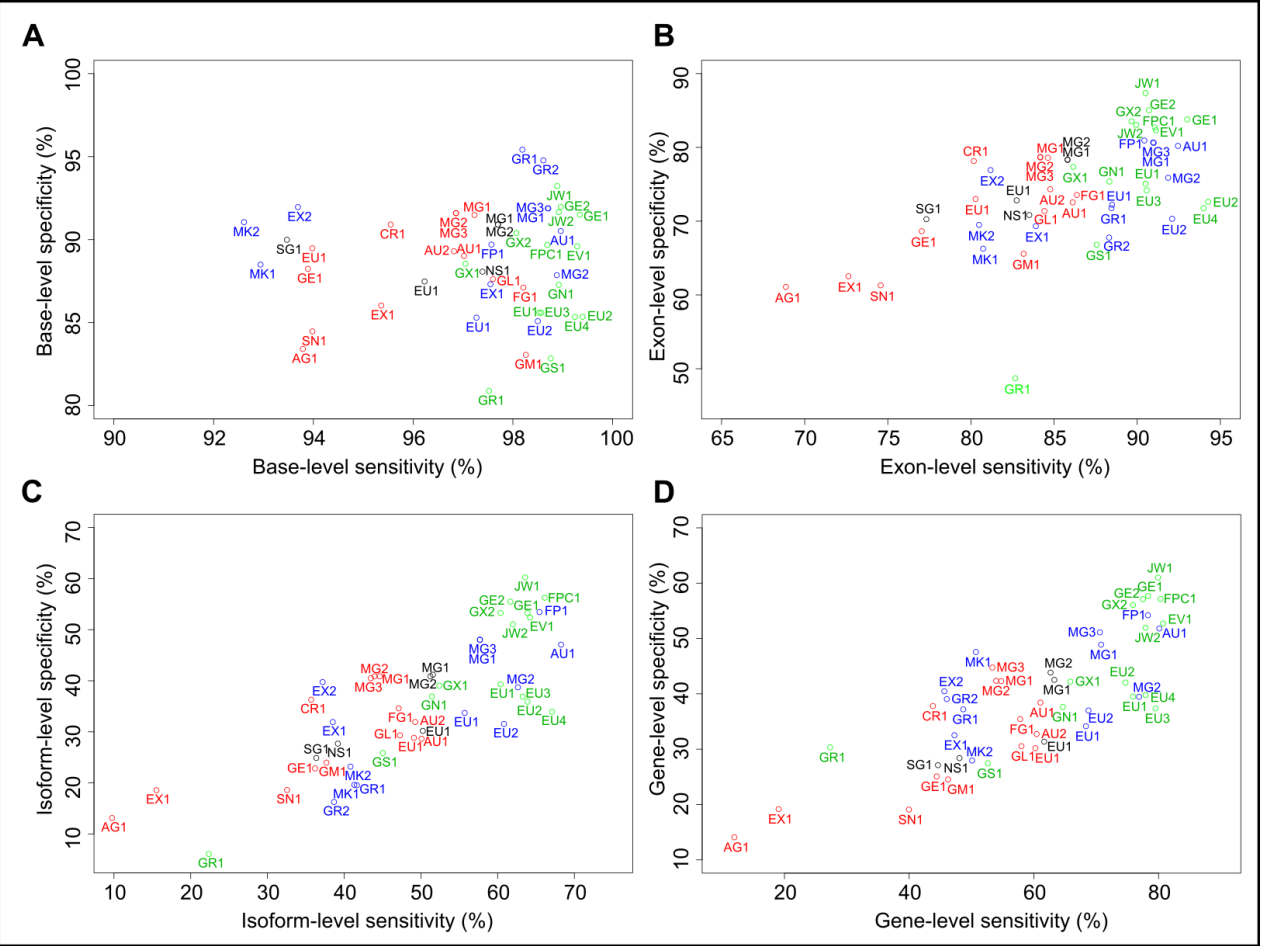
**Accuracy of the submitted gene sets**. Plots of the specificity against sensitivity of the submitted gene sets, at the base level (A), exon level (B), isoform level (C) and gene level (D). The submitted gene sets are coloured by nGASP category, with *ab initio *(category 1) gene sets in red, gene-finders that used multi-genome alignments (category 2) in black, gene-finders that used transcript/protein alignments (category 3) in blue, and combiners (category 4) in green. The gene sets are labelled as follows: AU: AUGUSTUS, MG: MGENE, CR: CRAIG, AG: Agene, EU: EUGENE, FPC: Fgenesh++C, FP: Fgenesh++, FG: Fgenesh, GE: GeneID, GM: GeneMark.hmm, GX: GENOMIX, GS: GESECA, GN: GLEAN, GL: GlimmerHMM, GR: Gramene, JW: JIGSAW, MK: MAKER (using SNAP), MG: MGENE, NS: N-SCAN, SG: SGP2, SN: SNAP, EX: ExonHunter, EV: Evigan.

The best submitted gene prediction sets had base level sensitivity in excess of 99% and specificity of more than 93% (Table 3; Figure 1). This means that the best gene predictors are able to identify almost all the protein-coding bases in the *C. elegans *genome and only occasionally predict that a non-coding base is coding. At the exon level, the best submitted gene sets had sensitivities of more than 91% and specificities of more then 83%. Thus, although most gene-finders identify most true coding bases correctly, they often do misidentify the boundaries of protein-coding exons. At the base and exon levels, specificities were lower than sensitivities. This may reflect a number of inaccurate gene models in the **ref2 **gene set, which included gene models not fully supported by transcript evidence, and perhaps also reflects exons that are missing from the **ref2 **gene set.

**Table 3:** Evaluation of submitted gene sets.

**Gene set**	**Category**	**Base**		**Exon**		**Isoform**		**Gene**	
		**Sn**	**Sp**	**Sn**	**Sp**	**Sn**	**Sp**	**Sn**	**Sp**

Agene	1	93.8	83.4	68.9	61.1	9.8	13.1	12.0	14.1

AUGUSTUS v1	1	97.0	89.0	86.1	72.6	50.1	28.7	61.1	38.4

AUGUSTUS v2	1	96.8	89.3	84.8	74.3	49.3	31.9	60.5	32.7

CRAIG	1	95.6	90.9	80.2	78.2	35.7	36.3	43.8	37.8

EUGENE	1	94.0	89.5	80.3	73.0	49.1	28.8	60.2	30.2

ExonHunter	1	95.4	86.0	72.6	62.5	15.5	18.6	19.1	19.2

Fgenesh	1	98.2	87.1	86.4	73.6	47.1	34.6	57.8	35.4

GeneID	1	93.9	88.2	77.0	68.6	36.2	22.8	44.4	25.1

GeneMark.hmm	1	98.3	83.1	83.2	65.6	37.7	24.0	46.3	24.5

GlimmerHMM	1	97.6	87.6	84.4	71.4	47.3	29.3	58.0	30.6

MGENE v1	1	97.2	91.5	84.6	78.6	44.6	40.9	54.8	42.3

MGENE v2	1	96.9	91.6	84.2	78.7	44.0	40.9	54.0	42.4

MGENE v3	1	96.9	91.6	84.2	78.6	43.5	40.5	53.4	44.8

SNAP	1	94.0	84.5	74.6	61.3	32.6	18.6	40.0	19.1

EUGENE	2	96.2	87.5	82.8	72.8	50.3	30.2	61.7	31.4

MGENE v1	2	97.7	90.9	85.8	78.4	51.6	41.2	63.3	42.5

MGENE v2	2	97.7	90.9	85.8	78.3	51.2	40.9	62.7	43.8

N-SCAN	2	97.4	88.1	83.5	70.8	39.2	27.7	48.1	28.4

SGP2	2	93.5	90.0	77.3	70.3	36.4	24.9	44.6	27.1

AUGUSTUS v1	3	99.0	90.5	92.5	80.2	68.3	47.1	80.1	51.8

EUGENE v1	3	97.3	85.3	88.5	72.2	55.7	33.7	68.4	34.2

EUGENE v2	3	98.5	85.1	92.1	70.3	60.8	31.5	68.8	36.1

ExonHunter v1	3	97.6	87.3	83.9	69.3	38.5	31.9	47.3	32.5

ExonHunter v2	3	93.7	92.0	81.2	76.9	37.2	39.7	45.6	40.5

Fgenesh++	3	97.6	89.7	90.4	80.9	65.5	53.4	78.3	54.2

Gramene v1^1^	3	98.2	95.4	88.5	71.8	41.7	19.6	48.7	37.2

Gramene v2^1^	3	98.6	94.8	88.3	67.8	38.7	16.3	46.0	39.0

MAKER (using SNAP) v1	3	92.9	88.5	80.7	66.3	41.3	19.6	50.7	47.6

MAKER (using SNAP) v2	3	92.6	91.1	80.5	69.5	40.8	23.2	50.1	28.0

MGENE v1	3	98.7	91.9	91.0	80.7	57.7	48.0	70.8	48.9

MGENE v2	3	98.9	87.9	91.9	75.9	62.6	38.7	76.9	39.5

MGENE v3	3	98.7	91.9	91.0	80.6	57.7	48.0	70.6	51.1

EUGENE v1	4	98.5	85.6	90.5	75.1	60.4	39.3	75.9	39.5

EUGENE v2	4	99.4	85.4	94.3	72.6	63.9	35.9	74.7	42.0

EUGENE v3	4	98.6	85.6	90.6	74.2	63.3	36.9	79.5	37.4

EUGENE v4	4	99.2	85.3	94.0	71.8	67.1	33.9	77.9	39.8

Evigan	4	99.3	89.6	91.1	82.3	64.2	52.4	80.7	52.7

Fgenesh++C	4	98.7	89.7	91.1	82.7	66.1	56.3	80.3	57.1

GeneID v1	4	99.3	91.5	93.0	83.8	63.9	53.3	78.3	57.7

GeneID v2	4	99.0	92.0	90.7	85.0	61.7	55.5	77.5	57.1

GENOMIX v1	4	97.1	88.6	86.2	77.4	52.4	39.0	65.9	42.2

GENOMIX v2	4	98.1	90.4	89.7	83.5	60.4	53.3	75.9	56.1

GESECA	4	98.8	82.8	87.6	66.8	45.1	25.9	52.6	27.4

GLEAN	4	98.9	87.3	88.3	75.4	51.4	37.0	64.7	37.6

Gramene^1^	4	97.5	80.9	82.7	48.7	22.4	6.1	27.3	30.3

JIGSAW v1	4	98.9	93.2	90.5	87.4	63.6	60.2	79.9	61.0

JIGSAW v2	4	98.9	91.7	89.9	83.0	62.0	51.1	77.9	52.0

^1^After the evaluations were complete, the GRAMENE developers discovered an error in their pipeline which incorrectly moved the end of the coding region by 3 bp in a significant fraction of their gene predictions, and this negatively affected the overall performance of GRAMENE.

The accuracy of the submitted gene sets evaluated using the reference gene sets ref1 and ref2. The sensitivity (Sn) results are given for reference set ref1, and the specificity (Sp) results are given for set ref2. The gene sets are divided according to nGASP category, where category 1 is *ab initio *gene-finders, 2 is gene-finders that used multi-genome alignments, 3 is gene-finders that used alignments of ESTs, mRNAS and proteins, and 4 is combiners.

The ultimate goal of a gene predictor is to predict entire genes correctly, including every alternative isoform. However, in practice gene-finders do not predict alternative isoforms of a gene very well. At the isoform level, the best gene sets had sensitivities of about 66% and specificities of about 56%. That is, the best gene-finders each missed about 34% of true *C. elegans *isoforms, indicating that gene-finders still need improvements in predicting alternative splice forms. At the gene level, the best submitted gene sets had sensitivities and specificities in excess of 80% and 58% respectively. That is, for 80% of genes in the **ref1 **reference set, the best gene predictors called at least one splicing isoform correctly across the entire length of its protein-coding region.

The isoform level is the most stringent level of assessment. However, given the low success of most gene-finders for predicting alternative splicing, gene level accuracy is generally considered more important for the purpose of annotating a newly sequenced genome such as that of *Caenorhabditis remanei*. That is, it is considered more important to predict at least one isoform of each gene correctly, rather than to predict all isoforms of one gene correctly and no isoforms of a second gene correctly. At the gene level, the most accurate gene-finders were combiners (Figure 1). Gene predictors that use alignments of ESTs, mRNAs and proteins came in second place. Combiners had higher sensitivity than algorithms that used expressed sequence alignments at the gene level (medians: combiners 78%, expressed sequence-based 68%, *P *= 0.04). However, in terms of specificity, there was no significant difference in gene level accuracy between combiners and gene predictors that used transcript and protein alignments (medians: combiners 42%, expressed sequence-based 39%, *P *= 0.1). Thus, by using diverse data such as expressed sequence alignments, multi-genome alignments and gene sets from different gene-finders, combiners improved the sensitivity of their predictions above those based on expressed sequence alignments alone. This agrees with EGASP [8], which reported that combiners had higher gene level sensitivities for human genes compared to gene-finders that used expressed sequence alignments alone (medians: combiners 70%, expressed sequence-based 64%) [8].

At the gene level, prediction algorithms that used expressed sequence alignments had higher sensitivity than *ab initio *gene predictors (medians: expressed sequence-based 68%, *ab initio *54%, *P *= 0.05), as well as higher specificity (expressed sequence-based 39%, *ab initio *32%, *P *= 0.01). This demonstrates that use of expressed sequence data leads to considerable improvements in the accuracy of gene-finders for *C. elegans*. This mirrors the findings of EGASP, which also reported higher gene-sensitivities for gene-finders that used transcript or protein alignments compared to *ab initio *gene-finders (medians: expressed sequence-based 63%, *ab initio *18%), as well as higher gene-specificities (expressed sequence-based 55%, *ab initio *8%) [8].

There was a tie for third place between gene prediction algorithms that used multi-genome alignments and *ab initio *gene-finders. The addition of multi-genome alignments to *C. briggsae *and *C. remanei *gave no statistically significant improvement in accuracy over *ab initio *predictions. This was surprising, as the EGASP project reported that gene-finders that used multi-genome alignments were more accurate than *ab initio *gene-finders for predicting human genes, in terms of both gene level sensitivities (medians: *ab initio *18%, multi-genome 26%) and specificities (*ab initio *8%, multi-genome 19%). This may reflect the relatively high gene level accuracy of *ab initio *gene-finders in *C. elegans *(medians: gene Sn 54%, Sp 32%), compared to human (Sn 18%, Sp 8%) [8], probably due to the compact nature of the *C. elegans *genome. In addition, it is possible that the evolutionary distances separating *C. elegans*, *C. briggsae *and *C. remanei *are less suited for inference of protein coding genes from multi-genome alignments than the corresponding set of vertebrate genomes used in the EGASP study. Furthermore, a difference in the way that the reference sets were defined for nGASP and EGASP could contribute to the observed difference in accuracy. For example, nGASP's use of different reference sets to estimate sensitivity and specificity might lead to different results compared to EGASP, which relied on a single set of reference genes to calculate both sensitivity and specificity.

In both nGASP and EGASP, the best gene-finders were combiners. However, in nGASP the median gene level sensitivity of combiners was 78% and specificity was 42%, while in EGASP the median gene level sensitivity of combiners was 70% and specificity was 52% [8]. In *C. elegans*, about 8% more of the true genes are predicted correctly, but 10% fewer of the gene predictions made are structurally correct. The lower specificity in *C. elegans *suggests that there are more real isoforms and/or real genes missing from the *C. elegans *curated gene set, compared to the human curated gene set. This could be due to the far smaller amount of transcript data available for *C. elegans *or more conservative manual curation of weakly supported isoforms or genes by the WormBase staff. Using the average of the sensitivity and specificity as an overall metric of accuracy, the *C. elegans *combiner gene sets were slightly less accurate (median 59%) than the human combiner gene sets (median 61%). However, it should be noted that some of the difference in accuracy between nGASP and EGASP may be due to the different test sets used.

In the results shown in Table 3, we calculated the accuracy of the phase 2 gene sets (combiners) in the 3' halves of the phase 1 test regions, while we calculated the accuracy of the phase 1 gene sets in the entire phase 1 test regions. To investigate whether this introduced a bias, we also calculated the accuracy of the phase 1 gene sets in the 3' halves of the phase 1 test regions. The sensitivity and specificity of the phase 1 gene sets were 1.5% and 0.6% lower on average in the 3' halves than in the entire phase 1 test regions (paired Wilcoxon tests: *P *= 10^-6^, *P *= 10^-5^). The source of this difference is not clear. However, as a result, when we look only at the 3' halves of the phase 1 test regions, the difference in accuracy between combiners and phase 1 gene sets is slightly greater than that shown in Table 3.

### Factors affecting gene-finding accuracy

**Figure 2:**
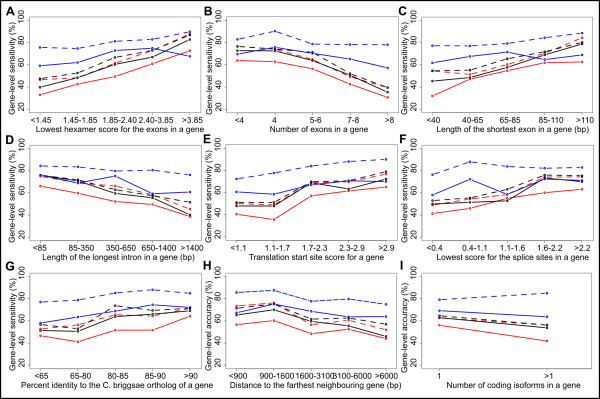
**Factors affecting gene-finding accuracy**. Plots of gene-level sensitivity against features of genes that are correlated with gene-finding accuracy: (A) the lowest hexamer score of any of the exons in the gene, (B) the number of exons in the gene, (C) the length of the shortest exon in the gene, (D) the length of the longest intron in the gene, (E) the strength of the translation start signal, (F) the lowest score of any of splice sites in the gene, (G) the percent identity with the *C. briggsae *ortholog at the amino acid level, (H) the maximum distance to a neighbouring gene, and (I) the number of isoforms in the gene. In each plot, the submitted gene sets are coloured by nGASP category, with *ab initio *(category 1) gene sets in red, gene-finders that used multi-genome alignments (category 2) in black, and gene-finders that used transcript/protein alignments (category 3) in blue. The solid lines show the median sensitivities of the gene sets in a category, while the dashed lines show the maximum sensitivity of the gene sets in a category.

To understand which factors affect the accuracy of gene-finders in *C. elegans*, we identified features of genes that were not predicted correctly by the *ab initio *gene-finders, gene-finders that used multi-genome alignments, and gene-finders that used expressed sequence alignments. The percentage of gene sets in which a true gene was predicted correctly (using the **ref1 **reference gene set) was found to be correlated with nine features of genes (Figure 2):

(i) the lowest 'hexamer score' of any of the exons in the gene (Spearman's *ρ *= 0.38, *P *< 10^-16^), using the score based on the frequency of 6-bp words from Genefeatures in the AceDB software [10],

(ii) the number of exons in the gene (*ρ *= -0.36, *P *< 10^-16^),

(iii) the length of the shortest exon in the gene (*ρ *= 0.30, *P *= 10^-11^),

(iv) the length of the longest intron in the gene (*ρ *= -0.29, *P *= 10^-9^),

(v) the strength of the translation start signal (*ρ *= 0.28, *P *= 10^-9^), as measured by Genefeatures,

(vi) the lowest score of any of splice sites in the gene (*ρ *= 0.25, *P *= 10^-7^), as measured by Genefeatures,

(vii) the percent identity with the *C. briggsae *ortholog at the amino acid level (*ρ *= 0.22, *P *= 10^-5^), based on an alignment from the TreeFam database of gene families [11],

(viii) the maximum distance to a neighbouring gene (*ρ *= -0.16, *P *= 0.0003), and

(ix) the number of isoforms in the gene (*ρ *= -0.11, *P *= 0.02).

That is, the *C. elegans *genes that are hardest for gene-finders to predict correctly are those with an exon of unusual hexamer content, lots of exons, a very short exon, a very long intron, a weak translation start signal, a weak splice site, a poorly conserved ortholog, as well as those that are far from their nearest neighbours, and those with many isoforms. We suggest that developers of gene-finding programs should concentrate on improving accuracy on these types of genes. The correlation with these features tended to be stronger for *ab initio *gene-finders than expressed sequence-based gene-finders (Figure 2). For example, the correlation with the lowest hexamer score for the exons in a gene was higher for *ab initio *gene-finders than for expressed sequence-based gene-finders (*ρ *= 0.42 and 0.22, Z-test: *P *= 0.0005). We observed weak or nonexistent correlations with other features that we examined, such as the length of the longest exon in a gene (*P *> 0.05), length of the shortest intron (*P *> 0.05), whether the adjacent genes are on the same strand (*P *> 0.05), existence of embedded genes with a gene's introns (*ρ *= -0.11, *P *= 0.01), whether a gene is member of an operon (*P *> 0.05), whether neighbouring genes are paralogs (inferred from TreeFam [11]; *P *> 0.05), and whether the gene overlaps a simple repeat or transposable element (*P *> 0.05).

There were 18 genes that were missed in all of the category 1, 2 and 3 gene sets, which must be the most difficult-to-predict: *C06G3.7 *(*trxr-1*), *C08G5.5*, *C33H5.14 *(*ntp-1*), *C55F2.1*, *D1009.1*, *F18E9.3*, *R04E5.7*, *R04E5.8*, *T07D3.4*, *T07F12.4*, *Y105E8A.7 *(*eat-18*), *Y43H11AL.1*, *Y48G8AL.7*, *Y54E5B.1 *(*smp-1*), *Y55F3BR.6*, *ZC455.6*, *ZC477.1 *(*ssq-3*), and *ZC8.4 *(*lfi-1*). Several of these genes have unusually long introns of > 1400 bp (*Y43H11AL.1*, *Y48G8AL.7*, *Y54E5B.1*), unusually short exons of < 40 bp (*D1009.1*, *Y105E8A.7*, *ZC8.4*), poorly conserved orthologs (*C08G5.5*, *R04E5.7*, *R04E5.8*), lots of exons (*F18E9.3, T07D3.4*), or are very far from one of their neighbours (*T07F12.4*).

### New gene sets for *C. remanei*, *C. brenneri*, *C. japonica *and *Brugia malayi*

To judge which gene-finders in each category performed best, we used the average of the gene level sensitivity and specificity for a gene set as a metric of overall accuracy. In collaboration with several of the nGASP contributors, we are assembling new gene sets for *C. elegans*, *C. briggsae*, *C. brenneri*, *C. remanei*, *C. japonica *and *Brugia malayi *using the three best performing of the gene-finders that used transcript/protein alignments: MGENE (Schweikert et al, submitted), AUGUSTUS[12] and FGENESH[13]. The best performing combiner, JIGSAW[14], is being used to combine the MGENE, AUGUSTUS and FGENESH predictions into a single nGASP gene set for each species that will form the basis of curated gene sets for the new genomes and will be used to improve curated gene models in *C. elegans*. All gene sets will be available from  and will also be displayed in the genome browsers for these species at .

## Conclusion

This experiment establishes a baseline of gene prediction accuracy in *Caenorhabditis *genomes, and is guiding the choice of gene prediction systems for the annotation of newly sequenced genomes for *Caenorhabditis *and other nematode species. At present, combiners are more accurate than other classes of gene prediction algorithms in *C. elegans*. However, the accuracy of the combiners would presumably benefit by increasing the accuracy of the component gene prediction sets that they are given. We have also identified features of *C. elegans *genes that are difficult to predict for *ab initio *gene-finders and gene-finders that use transcript-, protein- and multi-genome alignments, and hope that leaders in the gene prediction field will rise to the challenge of improving accuracy on such genes.

## Methods

### Data provided to the nGASP participants

#### Genomic DNA sequence

To select the nGASP test and training regions, we divided the WormBase WS160 *C. elegans *genome sequence into 102 non-overlapping regions of 1 Mb, and discarded regions of less than 1 Mb from the high-coordinate ends of the six chromosomes, leaving a set of 96 1 Mb regions. Representative training and test regions were selected from these regions based on gene density and conservation, following the strategy used to select the human ENCODE regions [8]. We measured gene density in each region by counting the number of curated genes, and assessed conservation with *C. briggsae *by using the number of bases covered by strong WABA [15] matches to *C. briggsae*. Regions were classified as having high or low gene density or conservation if their values lay in the top or bottom 33% percentiles respectively. The test and training sets each consisted of ten 1 Mb regions that were randomly chosen from the sets of regions with particular combinations of high/low gene density and high/low conservation (for example, we randomly chose two of the high conservation, low gene density autosomal regions; Table 2).

#### Auxiliary training data

We requested that gene-finders that had previously been trained using a large fraction of *C. elegans *confirmed genes or other data outside the supplied training sets be retrained solely on the training set provided by the nGASP project, namely:

(i) the coordinates of repeats found by RepeatMasker (A. Smit, unpublished, ) in the training regions.

(ii) the coordinates of coding exons, introns and UTRs in 584 confirmed isoforms (CDSs) of 432 genes in the training regions. An isoform was considered as confirmed if it was supported from start to end by mRNA, EST or OST transcript data.

(iii) the coordinates of coding exons, introns, and UTRs in 1583 'unconfirmed' isoforms of 1461 genes in the training regions. These genes lacked any confirmed isoforms.

(iv) the DNA sequence for the 'cb1' assembly of the *C. briggsae *genome.

(v) the DNA sequence for the 'pcap2' assembly of the *C. remanei *genome.

(vi) a multi-genome alignment between *C. elegans*, *C. briggsae *and *C. remanei *for the *C. elegans *training regions, made using MLAGAN version 1.21 [16].

(vii) the amino acid sequences of 42,496 proteins that have BLAST[17] matches to the test or training regions, excluding matches to proteins encoded by genes in the test regions. The BLAST matches were made by running BLAST with an *E*-value cut-off of 0.1 against proteins from *C. elegans *(wormpep160), *C. briggsae *(brigpep160), *Drosophila melanogaster *(FlyBase [18]), *Saccharomyces cerevisiae *(SGD [19]), UniProt [20], and human (Ensembl [21] and RefSeq [22]).

(viii) the nucleotide sequences of 20,141 *C. elegans *ESTs/cDNAs that have BLAT matches to the test or training regions.

(ix) the coordinates of the BLAST and BLAT matches in (vii) and (viii) in the test and training regions.

Participants were allowed to use different data for training, and for making predictions in the test regions, according to the nGASP category under which they were submitting a gene prediction set. The repeat sequences (i) and genes in the training regions (ii and iii) could be used by all participants. Participants who submitted *ab initio *(category 1) gene sets were not allowed to use any additional data for training or making gene sets. For gene-finders that used multi-genome alignments (category 2), participants could use the *C. briggsae *and *C. remanei *assemblies (iv and v) and the MLAGAN multi-genome alignment (vi). They also were allowed to generate a different multi-genome alignment using the tool(s) of their choice. For gene predictors that used expressed sequence alignments (category 3), participants could use the protein and transcript matches (vii, viii, ix), or they could choose a different alignment algorithm to realign the protein and transcript sequences contained in these sets.

For combiners (category 4), participants could use any of the auxiliary data allowed for categories 1–3, as well as the gene predictions submitted for categories 1 through 3 during nGASP phase one. Category 4 participants were also supplied with the coordinates of coding exons, introns, and UTRs in 386 confirmed isoforms of 242 genes in the 5' halves of each of the phase one test regions, which could be used as an additional training set. Because of this, combiners were evaluated using **ref1 **and **ref2 **gene sets drawn from the 3' halves of each phase one test region.

### Submission of gene sets

The submitted gene prediction files were required to be in GFF3 format (L. Stein, unpublished; ), an extension of GFF (Gene Feature Format; R. Durbin and D. Haussler; ). The GFF3 files were required to contain lines for gene, mRNA, CDS, and 5' and 3' UTR features. The format of gene prediction files submitted to nGASP was validated using a GFF3 format validator (P. Canaran, unpublished; ).

### Resources for assessing predictions: the reference gene sets

Predictions were compared to two different reference gene sets based on data in WormBase WS160 [4]: (i) all confirmed isoforms in the test regions ('**ref1**'), and (ii) all isoforms in all genes in the test regions ('**ref2**'). **Ref1 **consisted of 605 isoforms from 493 different genes, and **ref2 **consisted of 2250 isoforms from 1956 different genes. For phase two, we evaluated combiners using the 3' halves of each test region. The phase two **ref1 **and **ref2 **reference sets contained 313 isoforms from 249 different genes, and 1130 isoforms from 966 different genes, respectively.

We used **ref1 **to assess sensitivity and **ref2 **to assess specificity. This is because the true-positive and false-negative counts calculated by comparison to **ref1 **are more reliable than those calculated using **ref2**, as the gene models in **ref1 **are of higher quality. In contrast, the false-positive counts calculated by comparison to **ref2 **are more reliable, because a higher fraction of true genes are represented by gene models in **ref2**.

### Evaluation of accuracy of submitted gene sets

Two sets of evaluation software were written for nGASP. The first (P. Flicek, unpublished) was based on the earlier EGASP [8] evaluation software, but was extended to handle the GFF3 format for nGASP. The second software (A. Coghlan, unpublished) was written independently but calculated the same accuracy statistics.

### Data availability and visualisation

The nGASP test and training data, the submitted gene predictions and the command-line options and parameters used to generate them, and the **ref1 **and **ref2 **reference gene sets are available for download on the nGASP wiki  and on the nGASP ftp site .

**Figure 3:**
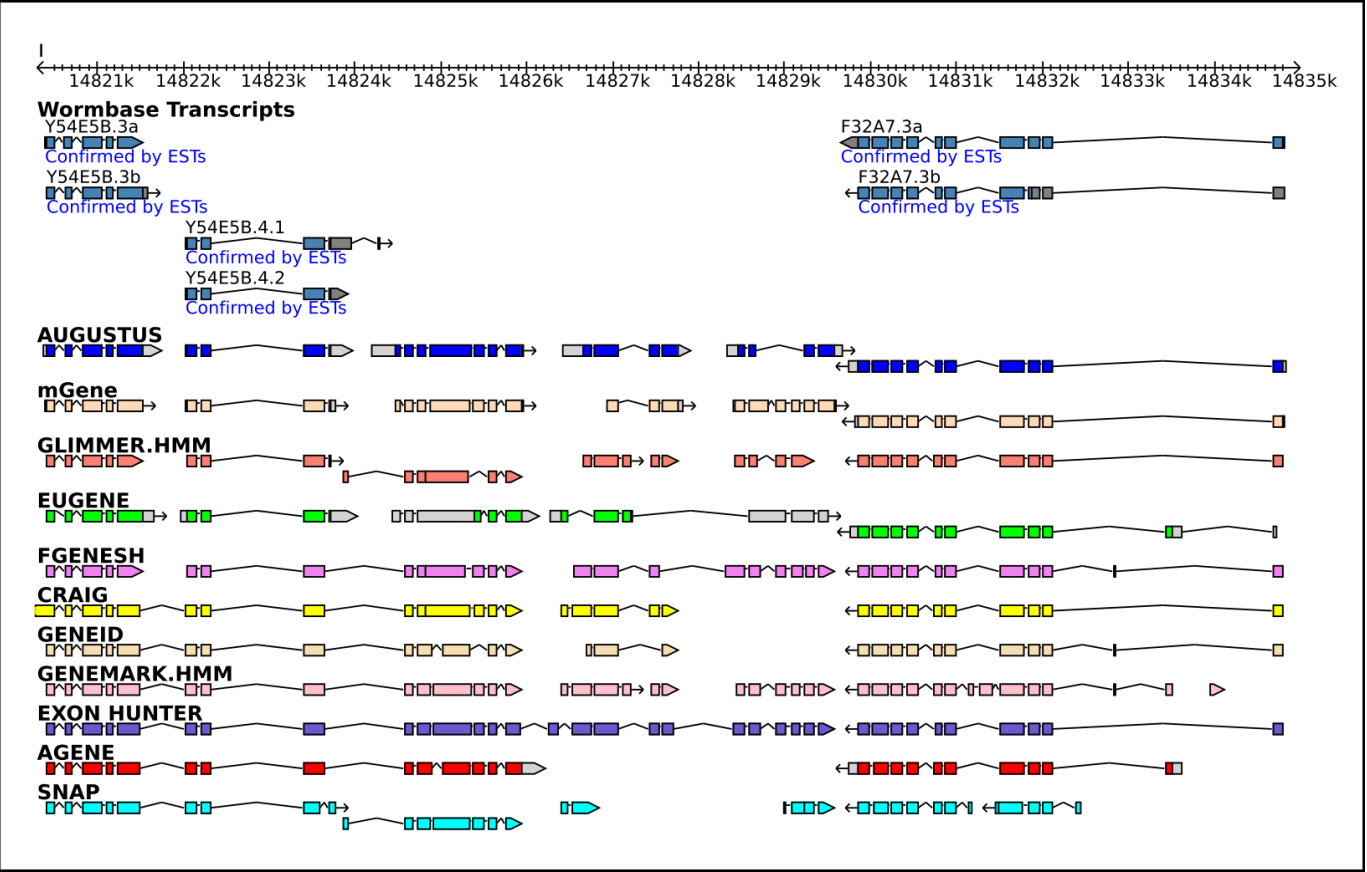
**A screenshot from the nGASP genome browser**. This shows part of an nGASP test region on chromosome I, with the curated WormBase gene models and the *ab initio *(category 1) gene sets submitted to nGASP for that region.

The submitted gene predictions and the reference gene sets can be viewed in a genome browser based on GBrowse [23] at . Each gene set is displayed in a different colour (Figure 3).

## Authors' contributions

TF, LS, AC, PF, SM and DB participated in the design of the study, and LS oversaw the study. TF and LS organised the competition and liaised with the groups that submitted gene sets to nGASP. The nGASP consortium submitted gene sets to nGASP. SM processed the raw submitted gene sets to ensure they were in standard GFF3 format. TH set up the ftp site, and LS and SM set up the genome browsers to display the nGASP gene sets. AC analysed the accuracy of the submitted gene sets, identified features correlated with gene-finders' accuracies, and drafted the manuscript. PF also analysed the accuracy of the submitted gene sets. DB and SM used some of the gene-finders that were judged most accurate by nGASP to produce new gene sets for the *C. elegans*, *C. briggsae*, *C. brenneri*, *C. remanei*, *C. japonica* and *Brugia malayi* genomes. Two groups in the nGASP Consortium (those of M. Stanke and G. Rätsch) made gene sets for the six nematode genomes using their gene-finders. SM set up an ftp site for gene prediction resources and the final nGASP gene sets for these species. All authors read and approved the final manuscript. For a list of the nGASP Consortium please see additional file 1

## Supplementary Material

Additional file 1 **The nGASP Consortium** Click here for file
