# Supplementary material for: nGASP – the nematode genome annotation assessment project
Source: BMC Bioinformatics. 2008 Dec 19;9:549. doi: 10.1186/1471-2105-9-549 (PMC2651883; doi:10.1186/1471-2105-9-549)
Supplement: Additional file 1 — The nGASP Consortium [file 1471-2105-9-549-S1.doc]

# The nGASP Consortium

geseca: Darin Blasiar^1^

n-scan: Randall H. Brown^2^, Michael R. Brent^2^

genomix: Avril Coghlan^3^, Richard Durbin^3^

GeneID and sgp2: Tyler Alioto^4^, Francisco Câmara^4^, Roderic Guigó^4^

snap: Ian Korf^5^

Gramene: Chengzhi Liang^6^, Doreen Ware^6,7^, Lincoln Stein^7^

GeneMark.hmm: Alex Lomsadze^8^, Vardges Ter-Hovhannisyan^8^, Andrey Kislyuk^8^, Mark Borodovsky^8^

Agene: Kasper Munch^9^, Anders Krogh^25^

Evigan and craig: Qian Liu^10^, Axel E. Bernal^10^, Fernando C. N. Pereira^10^, Aaron J. Mackey^11^, David S. Roos^11^

mgene: Gabriele Schweikert^12,13,14^, Georg Zeller^12,14^, Alexander Zien^12,15^, Jonas Behr^12^, Cheng Soon Ong^12,13^, Petra Philips^12^, Anja Bohlen^12^, Regina Bohnert^12^, Fabio De Bona^12^, Sören Sonnenburg^15^, Gunnar Rätsch^12^

glean: Aaron Mackey^11^, Qian Liu^10^, Fernando C. N. Pereira^10^, David S. Roos^11^

jigsaw: Jonathan E. Allen^26^, Steven Salzberg^16^

GlimmerHMM: Mihaela Pertea^16^, Steven Salzberg^16^

eugene: Jérôme Gouzy^17^, Céline Noirot^18^, Thomas Schiex^18^

Fgenesh, Fgenesh++, Fgenesh++C: Peter Kosarev^19^, Igor Seledsov^19^, Vladimir Molodtsov^19^, Victor Solovyev^19,20^

Augustus: Mario Stanke^21^

ExonHunter: Broňa Brejová^22^, Tomás Vinar^22^

maker: Brandi L. Cantarel^23^, Ian Korf^5^, Sofia M.C. Robb^24^, Genis Parra^5^, Eric Ross^24^, Barry Moore^23^, Carson Holt^23^, Alejandro Sánchez Alvarado^24^, and Mark Yandell^23^

^1^Washington University School of Medicine, St Louis, MO 63108, USA

^2^Laboratory for Computational Genomics, Washington University, Campus Box 8510, 4444 Forest Park Ave, St. Louis, Missouri 63108, USA

^3^Wellcome Trust Sanger Institute, Wellcome Trust Genome Campus, Hinxton, Cambridge, CB10 1SA, United Kingdom

^4^Bioinformatics and Genomics Program, Center for Genomic Regulation, Doctor Aiguader 88, E-08003, Barcelona, Spain

^5^Department of Molecular and Cellular Biology, University of California Davis, Davis, CA 95616, USA

^6^Cold Spring Harbor Laboratory, 1 Bungtown Rd, Cold Spring Harbor, NY, 11724, USA

^7^USDA-ARS NAA Plant, Soil & Nutrition Laboratory Research Unit, Cornell University, Ithaca, NY, 14853, USA

^8^The Wallace H Coulter Department of Biomedical Engineering and Computational Science and Engineering Division at College of Computing Georgia Tech Atlanta, Georgia 30332, USA

^9^Centre for Comparative Genomics, Department of Biology, University of Copenhagen, Universitetsparken 15, 2100 Copenhagen, Denmark

^10^Computer and Information Science Department. University of Pennsylvania, USA

^11^Department of Biology, Penn Genomics Institute. University of Pennsylvania, USA

^12^Friedrich Miescher Laboratory, Max Planck Society, Spemannstr. 39, 72076 Tübingen, Germany

^13^Max Planck Institute for Biological Cybernetics, Spemanstr. 38, 72076 Tübingen, Germany

^14^Max Planck Institute for Developmental Biology, Spemanstr. 35, 72076 Tübingen,

^15^Fraunhofer Institute FIRST.IDA, Kekulestr. 7, 12489 Berlin, Germany

^16^Center for Bioinformatics and Computational Biology, Biomolecular Sciences Building, University of Maryland, College Park, MD 20742, USA

^17^Unité de Biométrie et Intelligence Artificielle, INRA, UR 875, BP 52627, Chemin de Borde Rouge, 31326, Auzeville, France

^18^Laboratoire Interactions Plantes Micro-organismes UMR441/2594, INRA/CNRS, F-31Inc., 116 Radio Circle, Suite 400, Mount Kisco, NY,10549,USA

^19^Softberry Inc., 116 Radio Circle, Suite 400, Mount Kisco, NY,10549,USA

^20^Department of Computer Science, Royal Holloway, University of London, Egham, Surrey, TW20 0EX, United Kingdom

^21^Center for Biomolecular Science and Engineering, University of California Santa Cruz, USA

^22^Department of Biological Statistics and Computational Biology, Cornell University, Ithaca, NY 14853, USA

^23^Department of Human Genetics, Eccles Institute of Human Genetics, University of Utah, 15 North 2030 East, Salt Lake City, UT 84112-5330, USA

^24^Department of Neurobiology and Anatomy, Howard Hughes Medical Institute, University of Utah School of Medicine, Salt Lake City, UT 84132, USA

^25^Bioinformatics Centre, Department of Molecular Biology, University of Copenhagen, Ole Maaloes Vej 5, 2200 Copenhagen N, Denmark

^26^Lawrence Livermore National Laboratory, PO Box 808, L-174, Livermore, CA, 94551, USA

# 
